# Attention skills, learning and academic abilities in children and adolescents with genetic disorders: a systematic review

**DOI:** 10.3389/fpsyg.2025.1677418

**Published:** 2025-11-19

**Authors:** Elena Commodari, Valentina Lucia La Rosa, Francesca Foti

**Affiliations:** Department of Educational Sciences, University of Catania, Catania, Italy

**Keywords:** attention, learning, genetic disorders, children, adolescents

## Abstract

**Introduction:**

Attention plays a crucial role in learning by enabling concentration and retention. Deficits in attention are frequently associated with academic difficulties, particularly among children and adolescents with genetic disorders.

**Methods:**

A systematic review was conducted to examine the relationship between attention and academic performance in individuals with Down syndrome (DS), Fragile X syndrome (FXS), Neurofibromatosis Type 1 (NF1), Klinefelter syndrome (KS), Prader–Willi syndrome (PWS), Turner syndrome (TS), and Williams syndrome (WS). Eligible studies were identified through comprehensive database searches, and inclusion criteria focused on empirical research assessing both attention and academic outcomes. The review protocol was registered in PROSPERO (Registration No. CRD42024568993).

**Results:**

Nine studies met the inclusion criteria, focusing primarily on DS, FXS, and NF1. No eligible studies were found for KS, PWS, TS, or WS. Across conditions, attention deficits were significantly associated with lower academic performance, although patterns varied by disorder. In NF1, persistent attention and inhibitory control deficits were linked to impairments in reading and math. Single studies on DS and FXS showed similar associations that warrant further replication.

**Discussion:**

Although the available evidence is limited, the findings suggest that attention plays a critical role in academic achievement among individuals with genetic disorders. Future research should expand the evidence base and develop targeted interventions to address attention deficits and support learning outcomes in these populations.

**Systematic review registration:**

PROSPERO, CRD42024568993.

## 1 Introduction

### 1.1 An overview of attention skills

Attention is a critical component of the learning process that directly influences knowledge acquisition and application (Commodari and Guarnera, 2005). This function is important for achieving and maintaining a state of alertness, focusing and selecting specific sensory events as well as regulating thoughts and reactions in a targeted and goal-oriented manner (Klein et al., 2024; Petersen and Posner, 2012; Pozuelos et al., 2014). Efficient attentional processes allow children to focus on pertinent stimuli while disregarding distractions, thereby promoting the development of complex cognitive skills, including problem-solving, decision-making, and comprehension (Commodari, 2013; Commodari and Guarnera, 2005; Posner and Rothbart, 2007).

The framework proposed by Posner and Petersen conceptualizes attention as consisting of three partially independent yet functionally interconnected networks: alerting, orienting, and executive control networks (Commodari, 2017; Petersen and Posner, 2012). The *alerting* network regulates arousal and readiness to respond to stimuli. It encompasses both *tonic alerting*, which is maintaining alertness without external cues, and *phasic alerting*, which are transient increases in responsiveness to external cues. These abilities rapidly mature in early childhood and continue to develop with age, allowing children to sustain their attention during daily activities and learning tasks (Commodari, 2013; Rueda, 2014; Rueda et al., 2004). The *orienting* network directs and shifts attention to relevant locations or stimuli, supporting target recognition and the flexible allocation of mental resources (Posner and Rothbart, 2014; Pozuelos et al., 2014). As children grow, they become increasingly capable of voluntarily orienting their attention. This skill is fundamental for play and structured academic tasks, such as reading (Commodari, 2016; Posner and Rothbart, 2014). Finally, the *executive control* network underlies higher-order cognitive processes, such as planning, inhibitory control, decision-making, and error detection, and it continues to mature in parallel with prefrontal cortex development throughout childhood and adolescence (Commodari, 2013; Mezzacappa, 2004; Posner and Rothbart, 2014).

Individuals exhibit different degrees of development, efficiency, and functioning in each of these networks (Mezzacappa, 2004), with effects on the main aspects of attention, including focused, sustained, selective, distributed (or divided), and alternating attention (Posner et al., 1988).

The ability to react appropriately to particular stimuli is referred to as *focused attention*. This ability is associated with the alerting network, specifically phasing alerting, and is evaluated through recognition tasks, including the Continuous Performance test (Homack and Riccio, 2006). The ability to maintain focus and remain engaged with a task or stimulus for an extended period of time without being easily distracted is defined as *sustained attention* or vigilance. This type of attention also refers to the alerting network, specifically tonic alerting, and is critical for tasks that require continuous monitoring, such as reading or listening to a lecture, where a drop in attention can lead to missing information. Sustained attention is typically assessed by presenting individuals with distractors while they focus on a specific area of space or look for a specific target (Burack et al., 2012). The ability to focus selectively, often called *selective attention*, involves the executive network’s capacity to inhibit interfering distractions and suppress unsuitable reactions (Commodari, 2017; Lezak, 2012). The evaluation of this ability typically involves tasks that assess conflict arising from various dimensions of a target stimulus, such as the well-known Stroop Test (Stroop, 1935). The simultaneous maintenance of multiple attentional focuses, known as *distributed or divided attention*, requires the effective functioning of all these networks. This attentional skill refers to the distribution of resources among different sets of inputs (Parasuraman, 1998) and is usually assessed by administering dual-task tests (Hirano et al., 2020). Finally, the ability to rapidly shift attentional focus (Parasuraman, 1998), defined as *alternating attention*, depends on the orienting network (Commodari, 2017) and concerns the ability to change focus and tasks as well as the capacity to disengage and reengage attention in response to environmental stimuli (Lezak, 2012). It is usually assessed using multiple-choice tasks such as cancelation tests (Kaplan, 2011) or multiple-object tracking tests (Doran and Hoffman, 2010).

### 1.2 Attention and learning processes

Attention and learning are two interdependent processes supported by overlapping neural mechanisms. Attention allows individuals to focus on relevant stimuli, suppress distractions, and stay engaged long enough to encode and consolidate information (Rothbart and Posner, 2001). In turn, learning involves acquiring and retaining knowledge through experience, study, or instruction. This process relies heavily on memory and executive functions.

At the neural level, neuroimaging studies have shown that the prefrontal and parietal cortices play key roles in attentional control and learning processes due to their shared activation patterns (Friedman and Robbins, 2022; Gazzaley and Nobre, 2012). Activity in the dorsolateral prefrontal cortex is associated with working memory efficiency (Lara and Wallis, 2015; Miller and Cohen, 2001), and the anterior cingulate cortex contributes to error detection and conflict monitoring, thus supporting adaptive learning through attentional adjustments (Botvinick et al., 2001).

Each component of attention contributes uniquely to learning processes, enhancing the efficiency and effectiveness of knowledge acquisition and retention. Posner and Petersen (1990) demonstrated that selective attention can improve learning by facilitating the encoding and retrieval of information. In addition, selective attention is influenced by both bottom-up (stimulus-driven) and top-down (goal-driven) processes, highlighting the complex nature of attentional mechanisms in learning contexts (Corbetta and Shulman, 2002). Moreover, the ability to maintain attention for a prolonged and sufficient time to carry out an activity is critical for acquiring new knowledge, connecting new knowledge with prior knowledge, and engage in creative thinking (Commodari, 2012). Divided attention, on the other hand, enables the simultaneous processing of multiple sources of information, which is essential for multitasking and complex problem-solving (Schumacher et al., 2001). Additionally, shifting attention - the ability to switch focus between tasks or mental frameworks - plays a significant role in learning by allowing individuals to adapt to new information and changing environments, facilitating flexible thinking and problem-solving (Monsell, 2003).

The relationship between attention and learning becomes even more evident when considering the contribution of the different component of attention in several types of learning. In associative learning, attentional control strengthens the formation of links between stimuli, enabling individuals to anticipate outcomes and behaviors (Schultz, 2015). In language learning, attention facilitates the association of words with objects through repeated observation and interaction (Yu and Smith, 2016). Similarly, in motor learning, attention regulates the acquisition and refinement of new movement patterns (Song, 2019; Wulf and Lewthwaite, 2016), and in perceptual learning, attention enhances the ability to discriminate subtle sensory differences (Kellman and Garrigan, 2009; Treisman and Gelade, 1980). Even observational learning, a key mechanism for acquiring social and cultural knowledge, relies heavily on attention to model the behaviors and actions of others (Bandura, 2002). Children learn by observing and imitating their parents, teachers, and peers, which requires focused attention to details, intentions, and consequences of actions (Rogoff, 2003). This ability to observe and imitate others is closely related to broader social functioning and emotion regulation. Attention not only facilitates learning from others, but also plays a critical role in managing social interactions and regulating emotions (Posner and Rothbart, 2007). In this regard, it has been shown that children’s social functioning, especially the quality of their bond with caregivers, is strongly linked to their attentional performance. Children with good social adjustment tend to show faster reaction times, better focus on stimuli, and a stronger ability to shift attention compared to their peers with social functioning profiles characterized by insecure relationships (Commodari, 2013). Furthermore, deficits in attention can lead to difficulties in understanding and responding appropriately to emotional expressions, which can negatively impact social interactions and interpersonal relationships (Climie et al., 2019).

### 1.3 The relationship between attention skills and school abilities

The connection between attention and learning is especially clear in educational settings, where attentional control is essential for developing reading, writing, and math skills (Commodari, 2012, 2017; Commodari and Di Blasi, 2014; Commodari and Guarnera, 2005). Attention deficits can hinder academic performance by limiting a child’s ability to focus, follow instructions, and complete complex tasks (Polderman et al., 2010; Posner and Rothbart, 2014).

Attention deficits in reading hinder text tracking, decoding, and comprehension, resulting in slower reading speed and lower accuracy (Commodari, 2012; Vellutino et al., 2000; Vellutino et al., 2004). In writing, sustaining attention is essential for producing coherent text, retrieving words, and organizing syntax. Children with attentional difficulties often produce writing that is less structured, with frequent spelling and grammatical errors (Casas et al., 2011; García et al., 2014). Similarly, shifting attention efficiently between visual and motor inputs, such as when copying from the blackboard, is fundamental for error-free performance (Re and Cornoldi, 2015).

Mathematical reasoning and problem solving also depend on attentional control to follow multi-step procedures, inhibit irrelevant information, and sustain mental effort (Commodari and Di Blasi, 2014; Fuchs et al., 2005; Fuchs et al., 2006; Gallen et al., 2023; Orbach and Fritz, 2022). Difficulties in attention can lead to careless mistakes, slower computation, and reduced conceptual understanding. Over time, these deficits can accumulate, resulting in persistent academic challenges and lower educational attainment (Fredriksen et al., 2014). Therefore, early identification and targeted interventions are critical to preventing cascading effects on learning and motivation (Commodari and Di Blasi, 2014).

### 1.4 Attention skills and learning outcomes in children and adolescents with genetic disorders

Attentional and learning difficulties are particularly pronounced in children and adolescents with genetic disorders, who often experience increased distractibility, reduced sustained attention, and difficulties shifting focus (Kirk et al., 2017; Polderman et al., 2010). Understanding how attention influences learning in these populations is crucial for developing targeted educational strategies.

Several studies have investigated the relationship between attention and learning in children with Autism Spectrum Disorder (ASD) (Ames and Fletcher-Watson, 2010; Hanley et al., 2017; May et al., 2013; Mayes and Calhoun, 2007) or Attention-Deficit/Hyperactivity Disorder (ADHD) (DuPaul and Volpe, 2009; Faedda et al., 2019; Semrud-Clikeman and Bledsoe, 2011), while the literature on this topic in children and adolescents with genetic disorders is less extensive. This gap highlights the need for further investigation into the association between attentional skills and learning difficulties in these populations. Therefore, this review aimed to explore the attentional skills and learning abilities of children and adolescents affected by genetic disorders that are most frequently associated with attentional deficits and learning disabilities. In particular, we focused on the following genetic disorders, which we considered sufficiently representative of the major genetic conditions in developmental age: Down Syndrome (DS), Fragile X Syndrome (FXS), Klinefelter Syndrome (KS), Neurofibromatosis Type 1 (NF1), Prader-Willi Syndrome (PWS), Turner Syndrome (TS), and Williams Syndrome (WS).

DS is caused by a third copy of chromosome 21 instead of the standard two (Cenini et al., 2012), and its prevalence has recently been estimated to fall between 11.81 and 14,472 cases per 10,000 live births in the United States (Caban-Holt et al., 2015). DS is associated with various cognitive deficits, including attention and learning impairment (Foti et al., 2018). Children with DS have difficulties with sustained attention and working memory, which are crucial for learning complex tasks (Hasina et al., 2022; Lukowski et al., 2019).

FXS is a genetic condition that occurs because of either a full mutation or a highly localized methylation of the fragile X mental retardation 1 (FMR1) gene on the long arm of the X chromosome (Protic et al., 2022). FXS is the primary hereditary cause of intellectual disability and is frequently accompanied by significant attention deficits. Children with FXS often exhibit hyperactivity and impulsivity, and struggle to maintain their focus (Jordan et al., 2023).

KS affects approximately 1 in 650 males. It is characterized by one or more extra X chromosomes, usually 47 XXY, which leads to testicular dysfunction and can result in low fertility (Panvino et al., 2024). This genetic disorder is also associated with various cognitive impairments, including deficits in attention and executive functions. In particular, boys with KS often exhibit difficulties with sustained attention, working memory, and processing speed which affect their academic performance and overall learning abilities (Ross et al., 2008). These attentional challenges can lead to difficulties in organizing and completing tasks, following multi-step instructions, and maintaining focus during lessons, similarly to the other genetic disorders discussed (Samango-Sprouse et al., 2019).

NF1 is a genetic condition that affects both the central and peripheral nervous systems and occurs in approximately 1 in every 2,500 to 1 in every 3,000 individuals. Mutations in the neurofibromin 1 tumour suppressor gene located on chromosome 17 at position q11.2 are responsible for causing NF1, which can lead to a diverse range of symptoms (Crow et al., 2022). Most individuals affected by NF1 present multiple café au lait spots and cutaneous neurofibromas. Less common but more severe manifestations include central nervous system gliomas, plexiform neurofibromas, and malignant peripheral nerve sheath tumours (Rasmussen and Friedman, 2000). Although there is no consensus on a distinctive cognitive or behavioral profile for NF1, cognitive, neurobehavioral, and learning disorders are common (Acosta et al., 2012). NF1 is associated with low intellectual functioning, autism or other psychiatric disorders, motor delays, deficits in attention, executive function, and visuospatial skills. In addition, children and adolescents with NF1 often have learning difficulties and experience academic failure (Torres Nupan et al., 2017).

PWS is a genetic condition that occurs in approximately 1 out of every 15,000–20,000 births. It is typically caused by a deletion on the paternal chromosome 15q11-q13, which accounts for 70–75% of cases. In 20 to 25% of cases, it is caused by maternal disomy of chromosome 15, and in very rare cases (2%), it is caused by unbalanced translocations or imprinting centre defects (Whittington and Holland, 2017). PWS is associated with various cognitive impairments, including deficits in spatial domain as well as attention (Foti et al., 2015). These children often show sustained attention, working memory, and executive functioning challenges that hinder their academic performance (Foti et al., 2015).

TS, which affects approximately 1 in 2,500 females due to either the complete or partial absence of the second X chromosome with or without mosaicism (Hutaff-Lee et al., 2019), also poses challenges in attentional control. Girls with TS often experience difficulties with spatial attention and executive functions, which impacts their learning processes (Mauger et al., 2018).

WS is caused by a deletion in the long arm of chromosome 7 (Van Herwegen, 2015) and its incidence is approximately 1 in 7,500 live births (Stromme et al., 2002). WS is characterized by a unique cognitive profile with more preserved language skills, but significant deficits in visuospatial processing, executive functions, and attention (Foti et al., 2018). Children with WS often struggle with sustained attention and distractibility (Dominguez-Garcia et al., 2023).

In summary, understanding the specific attentional profiles and learning challenges of these genetic disorders is crucial in order to tailor educational interventions and support strategies to enhance learning outcomes in affected children. In light of these considerations, this systematic review aimed to synthesize the current research on attentional and learning abilities in these genetic disorders during development, highlighting the interplay between genetic factors, attentional control, and educational achievement.

### 1.5 The current study

Based on the existing reported literature, this systematic review aimed to comprehensively summarize and synthesize research investigating the association between attention skills and academic achievement in children and adolescents with some of the major developmental genetic disorders: Down syndrome, Fragile X Syndrome, Neurofibromatosis Type 1, Klinefelter Syndrome, Prader-Willi Syndrome, Turner Syndrome, and Williams Syndrome. In particular, the main objective of this study was to examine how attention deficits affect academic performance, including reading, writing, and math, in these populations. In summary, the main research question was: How do deficits in attentional skills (e.g., focused attention, inhibitory control) affect academic performance in children and adolescents diagnosed with the most common developmental genetic disorders?

## 2 Materials and methods

This systematic review was reported following the Preferred Reporting Items for Systematic Reviews and Meta-Analyses (PRISMA) guidelines (Page et al., 2021). The PRISMA checklist is available in the Supplementary materials. This systematic review has been pre-registered in the PROSPERO database with the registration ID: CRD42024568993.

### 2.1 Eligibility criteria

Studies were considered eligible for inclusion in this systematic review if: (a) the participants were children or adolescents between the ages of 6 and 18 years diagnosed with Down syndrome, Fragile X Syndrome, Neurofibromatosis Type 1, Klinefelter Syndrome, Prader-Willi Syndrome, Turner Syndrome, and Williams Syndrome; (b) the study design included cross-sectional or longitudinal assessments that examined attention skills and academic performance; (c) studies assessed at least one aspect of attention skills (e.g., focused attention, inhibitory control) and one aspect of academic achievement (e.g., reading, writing, math); (d) attention and academic achievement were measured using standardized instruments (e.g., Continuous Performance Test, Wechsler Individual Achievement Test); (e) studies included statistical analyses examining the association between attention skills and academic achievement. Only empirical, quantitative, and peer-reviewed studies were included; qualitative studies, review articles, short communications, editorials, and letters were excluded. Grey literature sources, such as conference abstracts, dissertations, or preprints, were not included to ensure methodological consistency and reliability of the synthesized evidence. There were no restrictions based on year or language of publication; if articles were written in a language other than English, professional translators would be used.

### 2.2 Search strategy

A comprehensive search strategy was used to systematically identify eligible research published in peer-reviewed journal articles. The literature search was conducted on 31 May 2024, updated on 12 October 2024, and finally on 7 October 2025 to ensure inclusion of the most recent publications. First, the bibliographic databases PubMed, Scopus, and PsycINFO were systematically searched. The following combination of keywords was applied in each database: (“attention” OR “attention skill*” OR “attention abilit*” OR “attentional control”) AND (“learning disabilit*” OR “learning difficult*” OR “learning disorder*” OR “academic performance”) AND (“child” OR “children” OR “adolescent*” OR “pediatrics”) AND (“neurofibromatosis 1” OR “NF1” OR “Down syndrome” OR “Trisomy 21” OR “Fragile X syndrome” OR “Turner syndrome” OR “Williams syndrome” OR “Klinefelter syndrome” OR “XXY Trisomy” OR “Prader-Willi syndrome”). The full search strings used in each database are available in the Supplementary materials. The reference lists of the included studies were further screened to identify any additional relevant papers. Covidence software (Veritas Health Innovation, 2021) was used to manage the searches and the screening procedure.

### 2.3 Data extraction

An Excel spreadsheet was created to extract the relevant information from the selected primary studies. All included studies were coded independently and simultaneously by two independent reviewers. Disagreements were resolved by discussion with a third reviewer until agreement was reached. The included articles were coded according to five dimensions: (1) study information (author(s), year of publication, country, study design, and exclusion criteria), (2) sample characteristics (e.g., sample size, age, genetic disorder, and gender distribution), (3) attention assessment (type of attention skills assessed and type of assessment instrument), (4) academic outcomes (type of outcome assessed: reading, writing, math performance, and type of assessment instrument), (5) main findings related to the relationship between attention skills and academic achievement.

### 2.4 Methodological quality assessment and risk of bias

The methodological quality and risk of bias of each study were assessed by the first author using the JBI Critical Appraisal Checklists for Analytical Cross-Sectional Studies, Case-Report Studies and Cohort Studies, which are widely used in the literature to assess the quality of different study designs included in systematic reviews. In addition, the second author independently assessed a subset of the selected studies (40% of the total, with a high percentage of agreement: 84%). Disagreements were resolved by discussion with a third reviewer until consensus was reached. Each JBI checklist includes items that assess key areas such as the inclusion of clear criteria for participants, the validity and reliability of exposure measures, the appropriate identification of confounders and the strategies used to manage them, and the assessment of outcome measures and statistical analysis.

Each item on the JBI checklist was answered with ‘yes’, ‘no’, ‘unclear’ or ‘not applicable’. To compare the quality of the studies between different designs, the percentage of positive responses (‘yes’) was calculated for each study. The studies were divided into three quality categories: low quality (less than 33% positive responses), medium quality (33–66% positive responses) or high quality (more than 66% positive responses). The full list of rating elements is available in the Supplementary materials.

## 3 Results

### 3.1 Study selection

**Figure 1:**
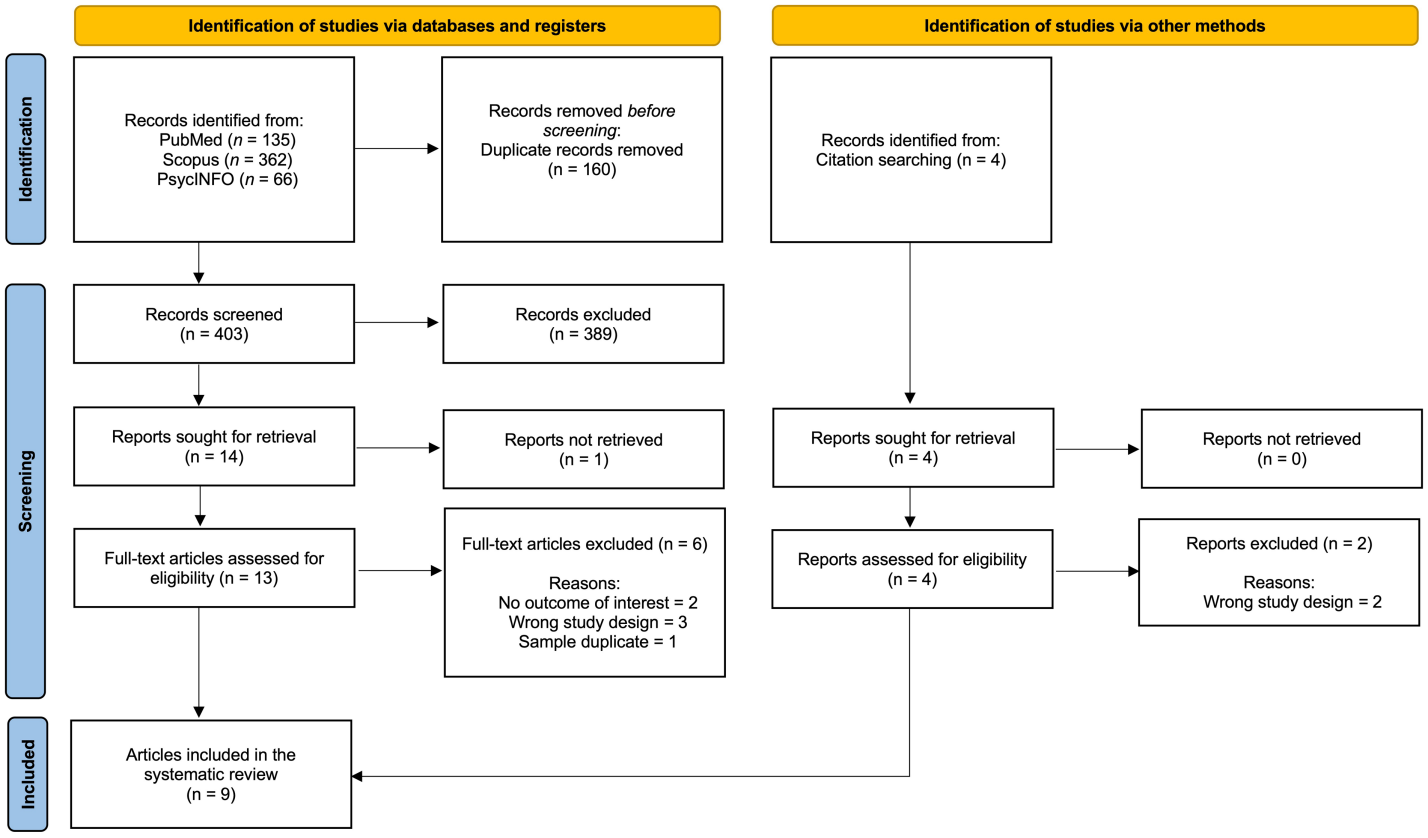
PRISMA flow diagram of included studies.

The results of the search strategies are shown in the PRISMA flowchart (Figure 1). A total of 567 abstracts were identified and 160 duplicates were removed. Two independent reviewers independently and simultaneously reviewed the remaining papers (*N* = 407). The percentage of agreement between the reviewers was substantial (Cohen’s Kappa = 0.78). Disagreements were resolved by discussion with a third reviewer, and final decisions were made after consensus was reached among all three reviewers. At this stage, 17 papers were selected. The full texts were then screened using the same procedure as for the abstracts, with a similarly high rate of agreement (Cohen’s Kappa = 0.74). In conclusion, 9 studies were included in this systematic review.

### 3.2 Study characteristics

Nine studies (Arnold et al., 2021; Chaix et al., 2018; Descheemaeker et al., 2005; Hou et al., 2023; Hyman et al., 2006; Janke et al., 2014; Jordan et al., 2023; Kirk et al., 2017; Siegel et al., 2024) were included in the systematic review. A summary of the main characteristics of the included studies is reported in Table 1. All articles were written in English. Regarding the year of publication, most of the studies (55.5%) were published recently, between 2018 and 2024 (Arnold et al., 2021; Chaix et al., 2018; Hou et al., 2023; Jordan et al., 2023; Siegel et al., 2024), while 44.5% were published between 2005 and 2017 (Descheemaeker et al., 2005; Hyman et al., 2006; Janke et al., 2014; Kirk et al., 2017). With regard to the study design, 44.4% adopted a case–control design (Arnold et al., 2021; Chaix et al., 2018; Hyman et al., 2006; Jordan et al., 2023), 33.3% of the studies used a cross-sectional design (Descheemaeker et al., 2005; Janke et al., 2014; Kirk et al., 2017), and 22.2% used a longitudinal design (Hou et al., 2023; Siegel et al., 2024). The total sample size across all studies was 757 participants (*M* = 84.1, SD = 40.5, range 17–150). Most samples included both male and female participants, with 375 females and 333 males. The average age of the study participants was approximately 10.75 years (SD = 1.75, range: 7–16.9 years). The studies analyzed several genetic disorders, primarily NF1, examined in 77.8% of the studies (Arnold et al., 2021; Chaix et al., 2018; Descheemaeker et al., 2005; Hou et al., 2023; Hyman et al., 2006; Janke et al., 2014; Siegel et al., 2024), DS (11.1%) (Kirk et al., 2017) and FXS (11.1%) (Jordan et al., 2023). No studies were selected that investigated variables of interest in the other genetic disorders under investigation. With regard to the geographical context, studies were mainly conducted in the United States (44.4%) (Hou et al., 2023; Janke et al., 2014; Jordan et al., 2023; Siegel et al., 2024) and Australia (33.3%) (Arnold et al., 2021; Hyman et al., 2006; Kirk et al., 2017); the remaining samples were from Europe (Belgium and France) (Chaix et al., 2018; Descheemaeker et al., 2005).

**Table 1:** Main characteristics of the included studies.

Author	Year	Country	Study design	Exclusion criteria	Genetic disorder	Sample size	Mean age	Study groups	Attention skills assessed	Assessment measures for attention	Academic outcomes assessed	Assessment measures for academic outcomes	Main findings
Descheemaeker et al.	2005	Belgium	Cross-sectional	Serious medical problems (e.g., optic pathway gliomas, other brain tumors, previous cranial irradiation, or epilepsy) IQ < 70	NF1	*N* = 17, *F* = 5, M = 12	9.2 years (range 7–11 years)	NF1 children with normal IQ (>70) and no serious medical issues	Sustained attention, inhibitory control, shifting	Stroop Color-Word Test Trail Making Test Bourdon-Wiersma Test	Reading (word recognition, comprehension), spelling, arithmetic (general and automated)	Real Word Reading Test Non-Word Reading Test Comprehensive Reading Test Spelling PDO-Praxis Kortrijkse Reken Test Tempo Test Arithmetic	There was no significant correlation between attention skills and academic performance (reading, spelling, arithmetic) in children with NF1.
Hyman et al.	2006	Australia	Case–control	Central nervous system pathologies (e.g., epilepsy, optic gliomas, brain tumors, hydrocephalus) Severe intellectual impairment Inadequate English proficiency	NF1	NF1: *N* = 81, *F* = 38, M = 43 Controls: *N* = 49, *F* = 29, M = 20	NF1: 11 years 6 months (range 8 years – 16 years 9 months) Controls: 12 years (range 8 years 2 months – 16 years 8 months)	81 children with NF1 (divided into subgroups: NF1-SLD, NF1-GLD, NF1-noLD) and 49 unaffected siblings	Sustained attention, selective attention, switching attention, divided attention	Test of Everyday Attention for Children Children’s Category Test Tower of London Controlled Oral Word Association Test	Reading, spelling, math, listening comprehension, oral expression	Wechsler Individual Achievement Test	Significant associations were found between attention deficits (sustained and switching attention) and lower academic performance in children with NF1. Children with SLD exhibited more severe attention deficits, particularly in sustained and divided attention, compared to those without learning disabilities.
Janke et al.	2014	USA	Cross-sectional	IQ < 70	NF1	*N* = 26, *F* = 14, M = 12	13.65 years (SD = 1.88)	26 adolescents with NF1	Inhibitory control	Delis-Kaplan Executive Function System (Trail Making, Color-Word Interference)	Word reading, spelling, numerical operations, reading comprehension	Wechsler Individual Achievement Test Second Edition (Word Reading, Reading Comprehension, Spelling, Numerical Operations)	Inhibitory control deficits were strongly associated with learning difficulties across all assessed academic areas. Inhibitory control was particularly crucial, contributing to both early math performance and reading skills.
Kirk et al.	2017	Australia	Cross-sectional	Visual, auditory, or motor impairments No elevated behavioral attention difficulties	DS	*N* = 77, DS: *F* = 10, M = 12; ASD: *F* = 4, M = 19; NSID: F = 14, M = 18	8.3 years (SD = 1.8)	77 children with ASD, DS, and NSID	Visual search, vigilance, inattention, hyperactivity	Wilding Attention battery Vigilance task	Phonological abilities, receptive vocabulary, cardinality (numeracy)	Phonological Abilities Test, Peabody Picture Vocabulary Test 4 Give-a-number protocol	Significant associations were found between visual attention difficulties and poorer academic performance in literacy (phonological abilities, vocabulary) and numeracy (cardinality) across all groups. Visual attention difficulties, particularly in children with DS, were strongly linked to academic weaknesses.
Chaix et al.	2018	France	Case–Control	A known neurological or psychiatric disorder (epilepsy, brain tumor, autism) IQ < 70 Uncorrectable hearing or visual impairment	NF1	*N* = 150, *F* = 78, M = 72	NF1: 10 years (SD = 1.3) Controls: 10 years (SD = 1.2)	75 children with NF1 and 75 matched controls	Sustained attention and inhibitory control	Connors Continuous Performance Test (omissions and commissions scores)	Reading	*L’Alouette* French Reading Test ODEDYS-2 test ORLEC battery	No significant differences were found between NF1 and non-NF1 children on mean attentional capacity. No significant differences were found between NF1 and non-NF1 children on reading comprehension and reading subskills (reading irregular words and pseudowords). Attention skills did not affect phonological and reading difficulties in the NF1 group.
Arnold et al.	2021	Australia	Case–Control	Intracranial pathologies (e.g., symptomatic optic gliomas) Vision or hearing loss Inadequate English proficiency IQ < 70	NF1	NF1: *N* = 60, *F* = 28, M = 32 Controls: *N* = 36, *F* = 18, M = 18	NF1: 8.75 years (SD = 1.84) Controls: 8.78 years (SD = −1.64)	60 children with NF1 and 36 typically developing Controls	Focused, sustained, alternating, and divided attention	Test of Everyday Attention for Children (Sky Search, Score, Creature Counting, and Sky SearchDT)	Word reading, reading comprehension, phonological decoding	Castles and Coltheart Word/Nonword test Test of Word Reading Efficiency Test of Everyday Reading Comprehension	Children with NF1 exhibited significant reading impairments compared to controls. Fewer inattentive behaviors were significant predictors of better word reading abilities. Visual attention deficits were linked to poorer reading performance.
Hou et al.	2023	USA	Longitudinal	- No plexiform neurofibromas (PNs) - Uncontrolled seizure disorders - Significant sensory deficits (e.g., hearing loss, blindness)	NF1	*N* = 88 F = 38 M = 50	12.05 years (3.62)	88 children with NF1 and plexiform neurofibromas	Sustained attention, inhibitory control	Connors Continuous Performance Test (omissions and commissions scores)	Math, reading, writing	Woodcock-Johnson Tests of Achievement-Third Edition (Letter-Word Identification, Reading Fluency, Passage Comprehension, Spelling, Writing Fluency, Writing Samples, Calculation, Math Fluency, Applied Problems)	Lower levels of inhibitory control were associated with a greater decline in reading scores over time, particularly in younger children. Attention showed a positive association with reading, along with working memory and cognitive flexibility.
Jordan et al.	2023	USA	Case–Control	Very preterm birth (<32 weeks) Significant sensory deficits Uncontrolled seizure disorder Psychosis, or bipolar disorder	FXS	*N* = 97, FXS: *F* = 55; Controls: *F* = 42	FXS: 10.6 years Controls: 10.4 years	Girls with FXS and age-, sex-, and verbal IQ-matched Controls	Inhibitory control, sustained attention	NIH Toolbox Cognition Battery (Flanker Inhibitory Control and Attention Test, List Sorting Working Memory Test)	Reading, math	KTEA-3 Brief (Letter & Word Recognition, Reading Comprehension, Math Concepts & Applications, Math Computation)	Girls with FXS showed significant deficits in attention and executive function compared to the control group. Deficits in sustained attention were significantly associated with poorer academic outcomes, especially in math. The attention-related deficits were not significantly correlated with reading performance in the FXS group.
Siegel et al.	2024	USA	Longitudinal	None specified	NF1	*N* = 76, *F* = 31, M = 45	12.75 years (SD = 3.36)	76 children and adolescents with NF1 and plexiform neurofibromas	Sustained attention, inhibitory control	Conners Continuous Performance Test (commissions, omissions and hit response times) Delis Kaplan Executive Function System (Trail Making subtest)	Spelling, writing mechanics, written expression	Woodcock-Johnson Tests of Achievement-Third Edition (WJ-III) Test of Written Language-4th edition	31.9% of the children scored below average in omissions (inattention), while 15.3% showed below average response times (Hit Response Time), indicating significant deficits in sustained attention. Measures of inattention were significantly associated with writing mechanics (Spelling, Writing Fluency, Contextual Conventions).

With regard to the assessment of attention skills, the most commonly used instruments were the Test of Everyday Attention for Children (Arnold et al., 2021; Hyman et al., 2006) and the Connors Continuous Performance Test (Chaix et al., 2018; Hou et al., 2023). With regard to the assessment of academic achievement, the Wechsler Individual Achievement Test (Hyman et al., 2006; Janke et al., 2014) and the Woodcock-Johnson Tests of Achievement (Hou et al., 2023; Siegel et al., 2024) were among the most widely used in the included studies to assess performance in reading, spelling, math and writing. All studies investigated the relationship between attention skills and school performance through statistical analyses.

### 3.3 Methodological quality and risk of bias of the studies

The results of the assessment of methodological quality and risk of bias are reported in Supplementary materials. All included studies were assessed as being of high quality, indicating an overall low risk of bias.

### 3.4 Summary of findings

In this systematic review, the selected primary studies examined a range of relationships between different components of attention and academic performance in children and adolescents with specific genetic disorders. In line with the main objectives of this review, the findings were grouped into three main categories based on the type of genetic disorder addressed in the selected studies: (a) NF1, (b) DS, and (c) FXS. For each genetic disorder, the main findings were discussed, focusing on the association between attention deficits (e.g., sustained attention, inhibitory control) and school performance (e.g., reading, writing, math), as well as the differences observed between age groups and the persistence and progression of these deficits over time.

#### 3.4.1 Associations between attention and academic outcomes in NF1

The seven studies reviewed (Arnold et al., 2021; Chaix et al., 2018; Descheemaeker et al., 2005; Hou et al., 2023; Hyman et al., 2006; Janke et al., 2014; Siegel et al., 2024) consistently highlighted the significant role of attention deficits, particularly sustained attention and inhibitory control, in the academic performance of children and adolescents with NF1. In almost all of these studies, attention deficits were closely associated with lower academic achievement in reading, writing, and math, although some differences were observed depending on the age of the participants and the school setting assessed.

Hyman et al. (2006), covering a wide age range (8 to 16 years), found that deficits in sustained and shifting attention were significantly associated with lower academic achievement in reading, spelling, and math. The study also found that children with NF1 and specific learning disabilities (SLDs) had more severe attention deficits, particularly in sustained and divided attention, than children with NF1 without SLDs.

Focusing specifically on preadolescents (mean age: 13.65 years), Janke et al. (2014) highlighted the importance of inhibitory control. In fact, deficits in inhibitory control were strongly associated with difficulties in several academic domains, including math and reading, highlighting the critical role of this attention skill in older children with NF1.

Arnold et al. (2021) reported similar findings in younger children (mean age: 8.75 years) with a specific focus on reading, identifying significant deficits in focused, sustained, alternating, and divided attention. More specifically, fewer inattentive behaviors were significant predictors of better word reading abilities, while visual attention deficits were linked to poorer reading performance, suggesting that attentional deficits significantly affect reading abilities.

In their longitudinal studies, Hou et al. (2023) and Siegel et al. (2024) showed that attentional deficits in children with NF1 worsened over time and significantly affected school performance. In particular, Hou et al. (2023) found that attention deficits worsened progressively, particularly affecting reading skills. Specifically, children with lower levels of inhibitory control experienced a more pronounced decline in reading scores with advancing age, highlighting the long-term consequences of attentional deficits on academic performance. Similarly, Siegel et al. (2024) reported that persistent deficits in sustained attention were significantly associated with poorer writing mechanics, such as spelling and writing fluency, over time. In their study, 31.9% of the children scored below average on measures of inattention, highlighting the high prevalence of attention difficulties in these children.

However, two other studies found no significant differences in the relationship between attention and academic achievement. Chaix et al. (2018) found no significant differences in attention span between children with NF1 and a control group of typically developing peers. Descheemaeker et al. (2005) also found no significant correlations between attentional skills (sustained attention, inhibitory control, and shifting ability) and academic performance in their sample of younger children (mean age: 9.2 years). This study reported no significant relationship between these attention deficits and school performance in reading, spelling, or math, which differs from the broader trend observed in other studies.

#### 3.4.2 Associations between attention and academic outcomes in DS

The only study included in the review of DS included mostly younger children (mean age: 8.3 years), with visual attention and vigilance as their main deficits. Kirk et al. (2017) compared children with DS, autism spectrum disorder (ASD), and non-specific intellectual disability (NSID) and found that children with DS had significantly poorer visual attention than their peers. These deficits were closely associated with poorer performance in phonological skills, receptive vocabulary, and numeracy, confirming the significant relationship between attention and learning outcomes in DS.

#### 3.4.3 Associations between attention and academic outcomes in FXS

Significant deficits in attention, particularly in sustained attention and inhibitory control, have been reported in children with FXS. Jordan et al. (2023), the only study of FXS included in this review, focused specifically on girls with FXS (mean age: 10.6 years) and compared them with typically developing peers of equivalent age, sex, and verbal IQ. Girls with FXS showed significant deficits in attention and executive function compared to the control group. Furthermore, the study found that attention deficits in the FXS group were more strongly related to lower math performance than to lower reading performance. The study suggests that attention deficits may have a domain-specific impact in late childhood, particularly in math, in girls with FXS.

## 4 Discussion

Attention is a fundamental cognitive skill that significantly influences learning processes at all stages of development (Petersen and Posner, 2012; Rueda, 2014). As discussed, it facilitates the ability to focus on relevant stimuli, ignore distractions, and maintain concentration, which are critical for the acquisition and retention of new knowledge (Song, 2019; Yu and Smith, 2016). The complex relationship between attention and learning is particularly evident in academic settings, where attentional control is required for tasks such as reading, writing and solving math problems (Commodari, 2012, 2017; Commodari and Di Blasi, 2014; Commodari and Guarnera, 2005). Attentional networks - alerting, orienting and executive control - work together to support different components of attention, such as sustained, selective and divided attention, all of which are essential for academic success (Geva et al., 2013; Rothbart and Posner, 2001; Rueda et al., 2004). Therefore, deficits in these attentional processes can lead to significant learning difficulties, particularly in children and adolescents.

In the context of genetic disorders, these attentional deficits are often exacerbated, contributing to more pronounced difficulties in academic performance. Genetic conditions, such as NF1, DS and FXS are often associated with specific attentional deficits, which negatively impact essential academic skills such as reading fluency, written expression and math problem solving, further complicating the educational experience of affected children (Hyman et al., 2005; Hyman et al., 2006; Lukowski et al., 2019; Munir et al., 2000; Tungate and Conners, 2021).

These findings are consistent with a broader body of evidence on attentional and executive function deficits in neurodevelopmental conditions such as ADHD. Meta-analytic reviews have demonstrated medium-sized impairments in working memory, response inhibition, and vigilance in individuals with ADHD (Pievsky and McGrath, 2018). These impairments are accompanied by alterations in fronto-striatal and fronto-parietal control networks involved in cognitive regulation (Cubillo et al., 2012; Mattfeld et al., 2014). Converging evidence from ADHD and genetic syndromes suggests that difficulties sustaining attention and inhibiting impulsive responses may reflect shared neurocognitive mechanisms rather than syndrome-specific anomalies. This transdiagnostic perspective emphasizes the central role of attentional control in learning and academic achievement, underscoring the importance of comparative and integrative research across neurodevelopmental disorders.

This systematic review provided a comprehensive overview of the relationship between attention deficits and academic achievement in children and adolescents with the main genetic disorders diagnosed at developmental age. Specifically, in line with our research question, the review highlighted the significant role that attention plays in academic achievement, particularly in literacy and numeracy, in individuals NF1, DS, and FXS, in order to interpret these findings within the broader context of the existing literature, and to consider the implications for educational interventions and future research directions.

### 4.1 Attention deficits in NF1 and academic achievement

Consistent with previous research, the majority of studies included in this review highlighted the impact of attention deficits, particularly sustained attention and inhibitory control, on academic outcomes in children and adolescents with NF1. Findings from studies such as Hyman et al. (2006) and Arnold et al. (2021) suggested that these attentional deficits have a profound effect on literacy and mathematical performance. For example, deficits in focused and divided attention are particularly detrimental to reading development, as children with NF1 struggle to maintain concentration on written material and to manage the visual attention tasks required for word decoding. These findings are consistent with models of reading acquisition that emphasize the importance of attentional resources for decoding and comprehension (Commodari and Guarnera, 2005; Posner and Rothbart, 2014). Furthermore, longitudinal evidence from Hou et al. (2023) and Siegel et al. (2024) suggested that these deficits not only persist but also worsen over time, particularly in relation to literacy. This finding supports that attentional control is a developmental process that, when impaired, can have long-lasting effects on educational trajectories. Importantly, the progression of these deficits calls for early intervention to mitigate the impact on academic achievement. However, not all studies have reported consistent results: Chaix et al. (2018) and Descheemaeker et al. (2005) did not observe a significant association between attentional skills and academic achievement, suggesting that the impact of attention on learning outcomes may vary depending on the specific attentional component assessed, the age of the participants, or the academic domain considered.

### 4.2 Attention deficits in DS and academic achievement

In contrast to NF1, only one study (Kirk et al., 2017) focused on the relationship between attention and academic outcomes in children with DS, highlighting the role of visual attention and vigilance deficits in their learning difficulties. The significant associations between poor visual attention and lower performance in phonological skills, receptive vocabulary and numeracy were consistent with previous findings that attention deficits exacerbate learning difficulties in children with DS (Faught et al., 2021; Lukowski et al., 2019). These data were consistent with the wider literature suggesting that children with DS often demonstrate impairments in executive function and working memory (Foti et al., 2018), both of which are critical for academic tasks requiring sustained and focused attention (Commodari, 2012).

The limited number of studies on DS in relation to attention and academic outcomes indicates a gap in the literature. Future studies should further explore the unique attentional challenges faced by children with DS and their impact on specific academic skills, particularly in older age groups, as attentional demands increase with the complexity of school tasks.

### 4.3 Attention deficits in FXS and academic performance

The only study included in this review that focused on FXS (Jordan et al., 2023) provided valuable insights into the domain-specific impact of attention deficits on academic performance in an all-female sample. Girls with FXS showed significant difficulties in sustaining attention and inhibitory control, which were strongly associated with lower performance in math. Interestingly, the study found that attention deficits had a greater impact on math than on reading, suggesting a possible domain-specific influence of attention in FXS. This finding is consistent with research showing that math performance is particularly sensitive to deficits in executive function and attentional control (Commodari and Di Blasi, 2014), which are critical for tasks requiring the processing of complex information and multistep procedures (Fuchs et al., 2005).

Given that FXS is one of the leading genetic causes of intellectual disability, it is crucial to further investigate the specific attentional challenges faced by this population. The findings suggest that tailored interventions targeting attentional control may be particularly beneficial for improving math outcomes in children with FXS, as well as providing insight into gender differences in attention-related learning difficulties.

### 4.4 Implications for educational interventions

The findings of this review highlighted the importance of addressing attention deficits in educational interventions for children and adolescents with genetic disorders. Given the pervasive impact of attention on academic performance, particularly in literacy and math, targeted interventions aimed at improving attentional control, such as cognitive training programs or classroom modifications, could significantly improve learning outcomes for these populations. Early identification of attentional deficits, followed by individualized support, is essential to mitigate the long-term impact on educational attainment.

In addition, the variability of findings across genetic disorders underscored the need for disorder-specific interventions that take into account the unique attentional profiles of each condition. For example, children with NF1 might benefit from interventions that focus on sustained attention and inhibitory control, whereas children with DS might require support with visual attention and working memory.

Beyond syndrome-specific adaptations, evidence from neurodevelopmental research suggests that attention training and executive function programs can substantially improve academic performance by enhancing cognitive regulation and goal-directed behavior (Diamond and Ling, 2016; Rueda et al., 2012). Classroom-based strategies, such as structured routines, multimodal instruction, environmental cues, and individualized scaffolding, can help children with genetic disorders manage attentional demands more effectively (Hanley et al., 2017). Incorporating these approaches within inclusive educational frameworks can reduce the attentional burden, promote active participation, and foster positive learning experiences.

Finally, interventions should be developmentally informed and culturally responsive. Attentional training and instructional design must consider the broader learning context, including cultural expectations, teaching styles, and available resources. Tailoring intervention programs to the specific cognitive and contextual needs of each child is essential to promoting equitable access to learning opportunities and supporting both academic and socioemotional development in children and adolescents with genetic disorders.

### 4.5 Limitations and future research directions

Despite the important implications of this systematic review for educational interventions targeting children and adolescents with genetic disorders, several limitations should be acknowledged.

A major limitation concerns the limited scope and representativeness of the available evidence. Only nine studies met the inclusion criteria, most of which focused on NF1, while a single study was found for DS and for FXS, and none for KS, PWS, TS, or WS. The evidence on DS and FXS therefore remains preliminary and should be interpreted with caution until replicated in larger and more diverse samples. The only study on FXS (Jordan et al., 2023) included an all-female sample, further limiting the generalizability of the results and highlighting the need to explore possible gender-related differences in attentional functioning across genetic syndromes. This unequal distribution of research efforts reflects both the rarity of these conditions and the small sample sizes that typically characterize this field, which together constrain the external validity of current findings. Looking ahead, it is essential to conduct coordinated multicenter studies to enable cross-syndrome comparisons and advance a more comprehensive understanding of attentional and learning profiles in genetic disorders.

Moreover, most of the included studies adopted a cross-sectional design and were based on small samples composed primarily of children or early adolescents. Such design limitations restrict the ability to identify developmental trajectories or causal relationships between attention and academic performance. Longitudinal studies are therefore needed to understand the development of attentional processes over time, identify optimal intervention periods, and clarify long-term implications for learning outcomes.

Methodological heterogeneity and inconsistent reporting, especially for studies on NF1, represent another critical limitation. The included studies varied widely in the tools used to assess both attention and academic achievement, and few provided standardized effect sizes or confidence intervals. This variability prevented the estimation of pooled effects and the conduct of a quantitative meta-analysis. To address these gaps, a structured narrative synthesis was carried out in accordance with PRISMA guidelines. Nevertheless, future research should adopt standardized assessment protocols and transparent statistical reporting practices to facilitate quantitative synthesis and improve comparability across studies. Furthermore, publication bias could not be formally assessed due to the small number of eligible studies, which prevented the use of standard statistical procedures, such as funnel plots or Egger’s test.

Demographic characteristics also emerged as a source of bias. Most studies were conducted in Western contexts, particularly the United States and Australia, and included predominantly male participants. This imbalance limits the cross-cultural relevance of the findings and restricts the analysis of gender-specific effects. Future research should examine how attentional and learning difficulties may vary across genders, cultural settings, and educational systems, and ensure more balanced and diverse samples.

Another important factor to consider is the presence of potential confounding conditions that may affect attention and learning in children with genetic disorders. Co-occurring neurodevelopmental conditions, particularly ADHD and ASD, are highly prevalent in several of the examined syndromes and may contribute to variability in cognitive and academic outcomes. For example, ADHD traits are often seen in children with NF1 or FXS, and ASD characteristics are common in children with DS or FXS (Torres Nupan et al., 2017). These comorbidities can exacerbate deficits in attentional control, executive functioning, and adaptive behavior, thus complicating the interpretation of syndrome-specific findings. Therefore, future studies should adopt designs that systematically assess and control for the presence of ASD and ADHD symptoms or explicitly examine their interactive effects on attention and learning outcomes.

Additionally, cultural and contextual factors may influence the development and evaluation of attentional abilities. Most of the studies included in this review were conducted in Western, English-speaking countries, where educational systems tend to emphasize individual performance, structured classroom routines, and standardized testing. These contextual characteristics may accentuate certain attentional profiles while masking others, limiting the cross-cultural applicability of current evidence. Comparative and cross-cultural research is necessary to understand how variations in educational practices, cultural expectations, and classroom environments influence attentional engagement and academic performance in children with genetic disorders.

Furthermore, the included studies did not comprehensively assess all components of attention. While some focused on sustained attention, others examined inhibitory control or shifting attention, but none provided a comprehensive assessment of the various attentional subcomponents. This partial assessment limits our understanding of how different aspects of attention contribute to academic performance. In addition, the use of different assessment tools across studies for both attention and academic outcomes has made it difficult to compare results and draw consistent conclusions. Standardized and comprehensive assessments using consistent tools across studies are needed to allow for more accurate comparisons and to better clarify how specific attention deficits affect learning in different genetic disorders.

Finally, future research should consider comorbid conditions that may exacerbate attentional difficulties in these populations, such as anxiety, hyperactivity, and executive dysfunction. Addressing these gaps will deepen our understanding of the complex relationship between attention and academic achievement in children and adolescents with genetic disorders. It will also guide the design of more targeted educational and clinical interventions.

## 5 Conclusion

In conclusion, this systematic review provides strong evidence of the significant impact of attention deficits on academic performance in children and adolescents with genetic disorders. The findings highlight the need for early and tailored educational interventions that address the specific attentional challenges faced by these populations. By better understanding the relationship between attention and learning, educators and clinicians can develop more effective strategies to support the academic success of children with genetic disorders, ultimately improving their quality of life and educational outcomes.

## Data Availability

The original contributions presented in the study are included in the article/Supplementary material, further inquiries can be directed to the corresponding author.
